# HSV-1 infection of human corneal epithelial cells: Receptor-mediated entry and trends of re-infection

**Published:** 2010-11-20

**Authors:** Arpeet Shah, Asim V. Farooq, Vaibhav Tiwari, Min-Jung Kim, Deepak Shukla

**Affiliations:** 1 Department of Ophthalmology and Visual Sciences, College of Medicine, University of Illinois at Chicago, Chicago, IL; 2 Department of Microbiology and Immunology, College of Medicine, University of Illinois at Chicago, Chicago, IL

## Abstract

**Purpose:**

The human cornea is a primary target for herpes simplex virus-1 (HSV-1) infection. The goals of the study were to determine the cellular modalities of HSV-1 entry into human corneal epithelial (HCE) cells. Specific features of the study included identifying major entry receptors, assessing pH dependency, and determining trends of re-infection.

**Methods:**

A recombinant HSV-1 virus expressing beta-galactosidase was used to ascertain HSV-1 entry into HCE cells. Viral replication within cells was confirmed using a time point plaque assay. Lysosomotropic agents were used to test for pH dependency of entry. Flow cytometry and immunocytochemistry were used to determine expression of three cellular receptors - nectin-1, herpesvirus entry mediator (HVEM), and paired immunoglobulin-like 2 receptor alpha (PILR-a). The necessity of these receptors for viral entry was tested using antibody-blocking. Finally, trends of re-infection were investigated using viral entry assay and flow cytometry post-primary infection.

**Results:**

Cultured HCE cells showed high susceptibility to HSV-1 entry and replication. Entry was demonstrated to be pH dependent as blocking vesicular acidification decreased entry. Entry receptors expressed on the cell membrane include nectin-1, HVEM, and PILR-α. Receptor-specific antibodies blocked entry receptors, reduced viral entry and indicated nectin-1 as the primary receptor used for entry. Cells re-infected with HSV-1 showed a decrease in entry, which was correlated to decreased levels of nectin-1 as demonstrated by flow cytometry.

**Conclusions:**

HSV-1 is capable of developing an infection in HCE cells using a pH dependent entry process that involves primarily nectin-1 but also the HVEM and PILR-α receptors. Re-infected cells show decreased levels of entry, correlated with a decreased level of nectin-1 receptor expression.

## Introduction

Herpes simplex virus (HSV) is a member of the alphaherpesvirus subfamily and has the ability to cause several ocular infections [1-3]. Primary infection of the virus spreads from cutaneous lesions or infections of mucosal surfaces to neuronal cell bodies, establishing a latent, lifelong infection. Specifically, herpes simplex virus 1 (HSV-1) is the cause of over 95% of cases of ocular herpes [2]. Infection generally occurs unilaterally and it remains the leading cause of infectious blindness in developed nations, partly due to its ability to latently infect hosts for long periods of time [1,2]. More than 20,000 new cases of ocular HSV-1 infection and an additional 28,000 reactivations occur in the United States annually [2]. HSV-1 infection causes a variety of ocular diseases including blepharitis, conjunctivitis, epithelial keratitis, stromal keratitis, endotheliitis and iridocyclitis – some of which pose a severe visual threat to infected hosts [2,3].

The corneal epithelium represents one of the major host sites of infection for HSV-1 and may precede infection of other locations within the eye [2]. The epithelium is composed of several layers of cells that protect the cornea's deeper layers, notably the stroma, and thus lies in the dominant pathway of infection by exogenous virus. The cornea is also the most highly innervated tissue in the body, facilitating the development of latency in trigeminal ganglia via retrograde transport of HSV. Some authors suggest that the cornea itself may be a site of latency, predisposing patients to increased morbidity resulting from localized viral reactivation [4-6]. Its continuity with the conjunctival epithelium further aides in the spread of virus in ocular infection [4]. While having such a critical role in ocular HSV, little is known of the mechanism of HSV-1 entry into human corneal epithelial cells.

This issue is particularly significant due to the potential for corneal infection to cause visual morbidity [7]. While epithelial keratitis can cause acute symptoms it also predisposes to stromal keratitis, which in turn can lead to scarring and opacification despite treatment [7,8]. While a minority of patients with initial ocular herpes infection present with stromal keratitis, it is much more frequent in the recurrent form of the disease and accounts for a significant portion of patients who develop blindness [1,2]. Thus, prevention of epithelial infection and its subsequent sequelae could improve the visual prognosis of patients.

Penetrating keratoplasty remains the most successful and most commonly practiced form of human tissue transplantation [9]. HSV keratitis is an important indication for corneal transplantation and is also a cause of graft failure [10]. There have been rare reports of donor-host transmission of HSV, which may be related to corneal latency [11]. It is suggested that the transplant procedure itself may be able to trigger latent virus to reactivate [12]. Potential complications following the procedure include recurrent herpetic keratitis and secondary non-viral infection [9,13,14]. Although the success of penetrating keratoplasty in HSV keratitis has improved, understanding the mechanism of infection is important as future treatment options are investigated.

Significant knowledge of the molecular mechanisms of HSV-1 infection in general has been obtained in previous studies. Infection of HSV-1 into cells involves multiple cell receptors and helper proteins found on the virion; these receptors and proteins aid in binding, fusion and entry into the target cell. At least five envelope glycoproteins are involved in entry: gB, gC, gD, gH and gL [15]. Viral glycoproteins gB and gC bind to the ligand receptor heparan sulfate which allows the virus to attach to the cell [16]. Next, a conformational change in the viral glycoprotein structure allows glycoprotein D (gD) to attach to its receptor (the gD receptor) located on the target cell [15]. Fusion of the viral envelope with the target membrane then occurs with the combined effects of gD, the gD receptor, gB, gH, gL, and possibly the gH coreceptor [15,17]. Once this series of events is initiated, fusion of the virion with the plasma membrane can occur via a pH independent process. In some cell types, a pH dependent pathway has been described with an endocytic mode of entry [18].

The importance of the gD receptors in entry is therefore a result of the role of gD as a catalyst. Among these, nectin-1 (HveC) and nectin-2 (HveB) belong to a distinct class of receptors in the immunoglobulin superfamily. Nectin-2, however, only aids in the entry of generated mutant strains of HSV-1 and not wild-type strains of the virus [15]. Herpesvirus entry mediator (HVEM) also associates with gD and belongs to the tumor necrosis factor receptor family [19]. 3-O sulfated heparan sulfate is a rare modified form of heparan sulfate that acts as a gD receptor for HSV entry into certain cell types and has antithrombotic properties [20]. paired immunoglobulin-like 2 receptor alpha (PILR-a) has been suggested to act as an entry receptor, but recent data suggests it may act more as a co-receptor [21].

While infection of the corneal epithelium is clinically important, little is known about how HSV-1 enters into corneal epithelial cells despite improved knowledge of neuronal reactivation [22]. It is also unknown to what extent virus-cell interactions in the corneal epithelium play a role in determining the course of disease. This study aims to determine the receptors that facilitate HSV-1 entry into human corneal epithelial cells and determine whether entry is pH dependent. After identifying the major molecular and cellular mechanisms of infection, this study aims to investigate re-infection of corneal epithelial cells and determine whether exposure to virus affects the ability of secondary infection to occur at early time points. The study then aims to determine if receptor expression is altered during re-infection.

## Methods

### Cells, viruses, and antibodies

The human corneal epithelial (HCE) cell line (RCB1834 HCE-T) was obtained from Dr. Kozaburo Hayashi (National Eye Institute, Bethesda, MD) [23]. The cells were grown in Minimal Essential Media (MEM; Gibco-Invitrogen Corp., Rockville, MD) and supplemented with penicillin, streptomycin, and 10% fetal bovine serum (FBS).

The African green monkey kidney (Vero) cell line as well as the wild-type Chinese hamster ovary (CHO-K1) cell lines were obtained (Patricia G. Spear, Northwestern University, Chicago, IL) and cultured as previously decribed [19]. The viruses used in the study – HSV-1(KOS), HSV-1(KOS)804, HSV-1(KOS)gL86, and HSV-1(KOS)RFP – were also provided by Patricia G. Spear [19]. All virus stocks were kept at a low multiplicity of infection (MOI), propagated in Vero cell lines, and stored at −80 °C. Titers for all three of the viruses were determined using Vero cells as well.

Anti-nectin-1 antibodies used were monoclonal mouse anti-nectin-1 (37–5900; Zymed Laboratories, San Francisco, CA). The monoclonal mouse anti-HVEM antibody was obtained from Santa Cruz Biotechnology (sc-74089; Santa Cruz, CA) and the monoclonal rat anti-PILR- α was obtained from Dendritics (ddx0230; Lyon, France). The secondary antibodies FITC-conjugated anti-mouse IgG and FITC-conjugated anti-rat IgG were obtained from Sigma-Aldrich (St. Louis, MO).

### Viral entry assays

Viral entry assays were performed using both HCE cells and naturally-resistant CHO-K1 cells, the latter of which do not possess the known viral entry receptors. Cells were plated on a 96-well tissue culture dish at full confluency (approximately 2×10^4^ cells per well). Cells were then infected with serial dilutions of β-galactosidase expressing HSV-1(KOS)gL86 virus at a maximum MOI of 0.01 in a solution of 3% BSA (BSA) in phosphate buffered saline (PBS). After 6 h of infection, cells were washed with 1× PBS and then incubated with *o*-nitrophenyl- β-D-galactopyranoside (ImmunoPure ONPG; Pierce, Rockford, IL), thus allowing quantification of β-galactosidase expressing HSV-1(KOS)gl86 viral entry into each of the cell types. Measurements of activity were then obtained at an optical density of 410nm by a spectrophotometer (Molecular Devices, Sunnyvale, CA).

HSV-1 entry into HCE and CHO-K1 cells was then confirmed qualitatively by 5-bromo-4-chloro-3-indolyl-β-D-galactopyranoside (X-gal) assays. Monolayers of cells were plated in a 6-well tissue culture dish (approximately 1.5×10^6^ cells per well). Cells were again infected for 6 h using the HSV-1(KOS)gL86 virus. After the incubation period, cells were washed, fixed, permeabilized, and incubated with the X-gal substrate. The X-gal substrate forms a blue color with cells that contain β-galactosidase. Microscopy images were collected using a 20× objective from the inverted microscope (Aciovert 100M; Zeiss, Jena, Germany) with the software Slidebook, version 3.0.

### Time point plaque assay

Viral entry and replication were again confirmed using a time point plaque assay. Monolayers of HCE, CHO-K1, and Vero cells were plated on a 6-well tissue culture dish (approximately 1.5×10^6^ cells per well). Cells were infected at 0.01 MOI with HSV-1(KOS)804 virus or mock infected with 1× PBS for 2 h. After primary incubation, the viral or mock solution were replaced with the proper media for each cell type – MEM with 10% fetal bovine serum (FBS) and penicillin/streptomycin additives for HCE cells, Ham's F-12 medium with 10% FBS and penicillin/streptomycin additives for CHO-K1 cells, and DMEM with 10% FBS and penicillin/streptomycin additives for Vero cells. Cells were then incubated at 37 °C for a series of time points. At the time points of 0, 12, 18, 24, and 36 h, incubated cells were fixed using 100% methanol and subsequently stained with a solution of 1% crystal violet in 50% ethanol at room temperature. Cells were then washed three times with nanopure water and the number and size of plaques were assessed as a measure of viral replication. Plaque size was measured and plotted as an average of 100 plaques formed at each time interval. Plaques were not significantly formed by the naturally-resistant CHO-K1 cells and thus plaque sizes were not measured or plotted. Images of the plaques were taken using the 40× objective from the inverted microscope (Aciovert 100M; Zeiss) using the software Slidebook version 3.0.

### pH dependence of entry

HCE cells were plated on a 96-well tissue culture dish (approximately 2×10^4^ cells per well). Cells were then treated with serial dilutions of BFLA-1 (1 μM) or chloroquine (1 mM) and incubated for 1 hat 37 °C. Concentrations of BFLA-1 and chloroquine followed those of previous studies [24,25]. Cells were then treated with equal concentrations of HSV-1(KOS)gL86 virus. The viral entry assay was then performed and β-galactosidase activity was determined using the same protocol as described above.

### Isolation of RNA

RNA was isolated from the Human Corneal Epithelial cells and CHO-K1 cells with TRIzol® reagent (Invitrogen, Carlsbad, CA). Total RNA samples were purified by RNeasy Mini kit (Qiagen, Valencia, CA) according to the manufacturer’s manual and checked the quantity of RNAs using NanoDrop (Thermo Scientific, Wilmington, DE). The quality of RNA was evaluated by NanoDrop (Thermo Scientific) and warranted that the ratio (28S/18S) of high RNA quality were >1.5. Densitometry analysis was performed using the NIH ImageJ software (version 1.43) and data for relative intensity was plotted as a histogram.

### Gene expression analysis and verification by qRT–PCR

To examine the gene expression by quantitative RT–PCR, 10 µg of the RNA samples from HCE and CHO-K_i_ cells were converted to first strand cDNAs using the High-Capacity cDNA Reverse Transcription Kits (Applied Biosystems, Foster City, CA) following the manufacturer’s protocol. The cDNA was added into the Platinum SYBR Green qPCR SuperMix-UDG Master Mixes (Invitrogen, Carlsbad, CA) containing SYBR Green I fluorescent dye and Taq DNA polymerase. The mixtures were aliquoted into 96-well plate containing gene specific primer sets. The sequence of the target gene primers included *HVEM* primers: 5′-TCT CTG CTG CCA GAC A-3′ and 5′-GCC ACA GCA GAA CAG A-3′, Nectin-1 primers: 5′-TCC TTC ACC GAT GGC ACT ATC C-3′ and 5′-TCA ACA CCA GCA GGA TGC TC-3′, *PILR-α* primers: 5′-AAG GTC AGC AGC GGA CTA AA-3′ and 5′-CAG TCT TGA GAG GGC TGT CC-3′, beta-actin (*ACTB*) primers: 5′-GTG ATG GTG GGA ATG GGT CAG-3′ and 5′-TTT GAT GTC ACG CAC GAT TTC C-3′. The qRT–PCR reactions were performed using ABI Prism 7500 system (Applied Biosystems) with the following parameters; repeat 1 (1 cycle), 2 min at 50 °C; 2 min at 95 °C and repeat 2 (40 cycles), 15 s at 95 °C and followed by 30 s at 60 °C, and the data were collected at each 60 °C. PCR products were ran on a 1% agarose gel, stained with ethidium bromide and illuminated with UV light. Expected PCR gene product sizes were 1,270 bp for *HVEM*, 738 bp for nectin-1 and 250 bp for *PILR-α*.

### Immunocytochemistry

To visualize various receptors on the cell surface, monolayers of HCE cells were plated on chamber slides (Lab-Tek; Nunc, Rochester, NY) at 100% confluency. After a 1× PBS wash, cells were incubated with anti-nectin-1 (1:50 dilution), anti-HVEM (1:50 dilution), or anti-PILR-α (1:25 dilution) primary antibody for 90 min at 4 °C. After the primary incubation period, cells were washed three times with a cold solution of PBS containing 3% BSA for 10 min. Next, cells were incubated with secondary antibody. The secondary antibody used for the anti-nectin-1 and anti-HVEM primary antibody was FITC-conjugated anti-mouse IgG (Sigma) while the secondary used for anti-PILR-α antibody was FITC-conjugated anti-rat IgG (Sigma); all secondary antibodies were administered at a concentration of 1:1,000 and had an incubation period of 45 min at 4 °C. Cells were then washed three times with a cold solution of PBS containing 3% BSA for 10 min. Final products were imaged using laser-scanning spectrum confocal microscopy (TCS SP2; Leica, Wetzlar, Germany). HCE cells treated with only secondary antibody as well as primary antibodies replaced with isotypes served as negative controls. A monoclonal anti c-Myc IgG in generated in mouse and a rat anti c-Myc IgG (Abcam) provided the isotype controls.

### Flow cytometry

Flow cytometry was used to confirm the presence of viral entry receptors nectin-1, HVEM, and PILR-α on the cell surface visualized by immunocytochemistry. Monolayers of HCE cells plated on 6-well tissue culture dishes were transferred to 1.5 ml Eppendorf tubes using Hank's based enzyme-free cell dissociation buffer (Invitrogen) and were then washed three times for 10 min with a cold solution of 1× PBS with 10% glucose and 10% calf serum. Cells were then incubated at 4 °C for 90 min with either anti-nectin-1 (1:100 dilution), anti-HVEM (1:100 dilution), or anti-PILR-α (1:100 dilution) primary antibody. Cells were again washed three times for 10 min with a cold solution of 1× PBS with 10% glucose and 10% calf serum and then incubated for 45 min at 4 °C with the appropriate FITC-conjugated secondary antibody as noted above at a concentration of 1:1,000. After incubation of secondary antibody, the cells had a final wash for 10 min with a cold solution of 1× PBS with 10% glucose and 10% calf serum. Flourescence-activated cell sorter (FACS) analysis was then performed.

### Antibody blocking of receptors

HCE cells plated on a 96-well tissue culture dish (approximately 2×10^4^ cells per well) were treated with serial dilutions of the appropriate antibody (anti-nectin-1, anti-HVEM, or anti-PILR-α) to block their corresponding receptor. The dilutions of the antibodies used ranged from 1:25 to 1:1600. Following incubation at room temperature for 2 h, viral entry assays were performed using HSV-1(KOS)gL86 virus as per the protocol stated above. Cells not blocked with primary antibody were used as controls.

### Re-infection

HCE cells plated on a 96-well tissue culture dish (approximately 2×10^4^ cells per well) were treated with a primary viral dose of HSV-1(KOS) at a concentration of 0.01 PFUs/cell. After 48 h of incubation at 37 °C, cells were treated with a serial dilution of a secondary viral dose of HSV-1(KOS)gL86. Viral entry assays were subsequently performed as per the protocol above. Cells treated with only the secondary dose of virus served as controls. To determine the proportion of cells infected during the primary infection, cell staining was performed. HCE cells plated in a 6-well culture dish at full confluency were treated with a viral dose of HSV-1(KOS) at a concentration of 0.01 PFUs/cell. After 48 h of incubation at 37 °C, cells were rinsed with 1× PBS wash. Primary HSV-1 polyclonal antibody (Dako, Carpinteria, CA) was added at a 1:50 dilution and incubated for 1 h at room temperature. After a 1× PBS wash, endogenous peroxidase activity was blocked by adding 3% hydrogen peroxide for 10 min at room temperature. After another 1× PBS wash, secondary anti-HSV-1 antibody conjugated to horseradish peroxidase (1:200 dilution) was added and incubated for 30 min at room temperature. After a final wash, DAB was added. Cells infected with virus were stained brown. The proportion of infected cells in 100 high power fields were determined and averaged.

### Flow cytometry post-primary infection

HCE cells plated on a 6-well tissue culture dish were treated with a viral dose of HSV-1(KOS)RFP at a concentration of 0.01 PFUs/cell. After 48 h of incubation at 37 °C, the monolayers of cells were transferred to 1.5 ml Eppendorf tubes. Before FACS analysis was performed, cells were treated with propidium iodide to distinguish live from dead cells. Flow cytometry was then selectively performed to test levels of nectin-1 on living cells tagged with red flourescent protein as per the method described above. The concentration of anti-nectin-1 primary antibody was 1:100 and the concentration of anti-mouse IgG secondary antibody was 1:1,000. HCE cells untreated with a viral dose were used as a control.

### Statistical analysis

All of the experiments were conducted a minimum of 3 times with results at a steady level. Quantitative data was expressed as a mean±standard deviation.

## Results

### HSV-1 entry and replication in HCE cells

**Figure 1:**
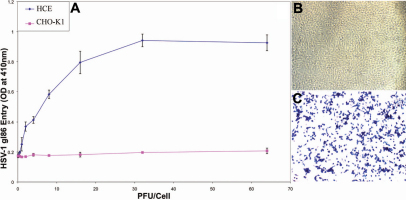
SV-1 can enter into culture HCE cells. **A**: Dose response curve of HSV-1 entry into HCE cells. Cultured HCE cells along with naturally HSV-1 resistant CHO-K1 cells were plated in 96-well culture dishes and inoculated with twofold serial dilutions of β-galactosidase-expressing recombinant HSV-1(KOS)gL86 virus at the plaque forming units (PFU) indicated. After 6 h, the cells were washed, permeablized and incubated with ONPG substrate. Viral entry was measured using a spectrophotometer which measured beta-galactosidase activity at an optical density of 410 nm. Values in the figure were plotted as the mean of three determinations (±SD). **B**: Confirmation of HSV-1 entry into HCE cells by X-gal staining. HCE cells grown (4×10^6^ cells) in six well dishes were inoculated with β-galactosidase-expressing HSV-1(KOS)gL86 virus at 20 PFU/cell. CHO-K1 cells were also infected in parallel as a negative control. After 6 h of infection at 37 °C, cells were washed, fixed, and permeabilized. X-gal was then added which yields an insoluble blue product upon hydrolysis by β-galactosidase. Blue cells, which represent cells with viral entry, were seen in HCE cells, but not the naturally resistant CHO-K1 cells. Microscopy was performed using a 20X objective of the Zeiss Axiovert 100 microscope. The slide book version 3.0 was used for the images.

As shown in Figure 1A, viral entry into HCE cells compared to that of CHO-K1 cells followed a dose-dependent pattern, with maximum entry occurring before maximum viral concentration. Upon establishing initial data on viral entry using these results, viral entry was verified using the insoluble substrate X-gal. In Figure 1B, β-galactosidase activity was not observed in the naturally resistant CHO-K1 cells, whereas β-galactosidase activity was observed in HCE cells (Figure 1C).

**Figure 2:**
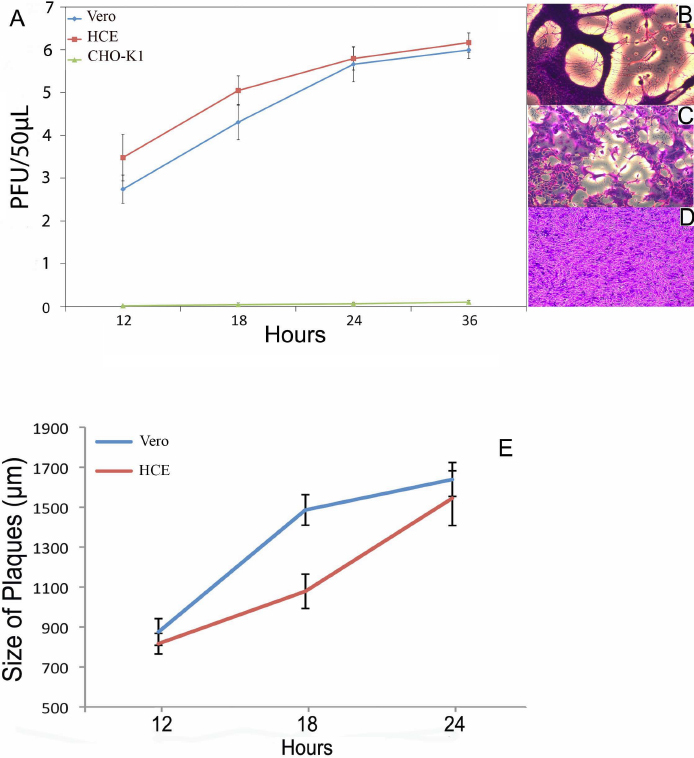
HSV-1 can successfully grow and replicate in HCE cells. Confluent monolayers of HCE, Vero, and naturally-resistant CHO-K1 cells were infected with HSV-1(KOS)804 at an MOI of 0.01 for 90 min at 37 °C. Innoculums were harvested, fixed, and stained at 0, 12, 18, 24, and 36 h. Data represents the mean of three samples (±SD). A typical plaque formed by Vero and HCE cells stained with crystal violet at 18 h of infection with HSV-1(KOS)804 virus is shown in (**B**) and (**C**) accordingly. Plaques were not formed in the HSV-1 resistant CHO-K1 and the lack of plaques are shown in **D**. Increasing plaque size over time demonstrating progressive infection was confirmed in **E**.

After establishing HSV-1 viral entry into HCE cells, we examined the ability of HSV-1 to successfully replicate within the cells using a time point plaque assay. As seen in Figure 2A, the number of plaques followed a time-dependent pattern for HCE cells and Vero cells (positive control). Image confirmation of plaques after 18 h post-infection in Vero and HCE cells are demonstrated in Figure 2B,C, respectively. Figure 2D confirms that plaques were not formed in the naturally resistant CHO-K1 cells at 18 h post-infection. Plaque size was also measured at the time intervals of 12, 18, and 24 h and plotted in Figure 2E. An increase in average plaque size at successive time points was observed indicating the ability of virus to spread to adjacent uninfected cells, which is also observed during a primary or recurrent infection in the cornea [26].

### pH dependency of viral entry

**Figure 3:**
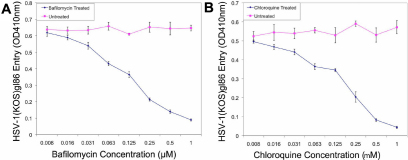
HSV-1 entry into HCE cells is a pH dependent process. Monolayers of HCE cells were either mock treated (no treatment) or treated with indicated concentrations of bafilomycin (**A**) or chloroquine (**B**) and exposed to HSV-1(KOS)gL86. Viral entry was measured using a spectrophotometer at an OD of 410 nm and plotted as the mean of three samples (±SD).

HSV-1 entry can be pH dependent or independent depending on cell-type [18]. Bafilomycin and chloroquine are both lysosomotropic agents that increase pH and therefore influence cellular uptake in pH dependent mechanisms (e.g., endocytosis). As shown in Figure 3, both bafilomycin and chloroquine strongly inhibited HSV-1 entry in a dose-dependent manner; when concentrations of these lysosomotropic agents were increased, measurements of viral entry decreased. This suggests that HSV-1 entry into HCE cells requires acidification.

### Identification and expression of entry receptors

**Figure 4:**
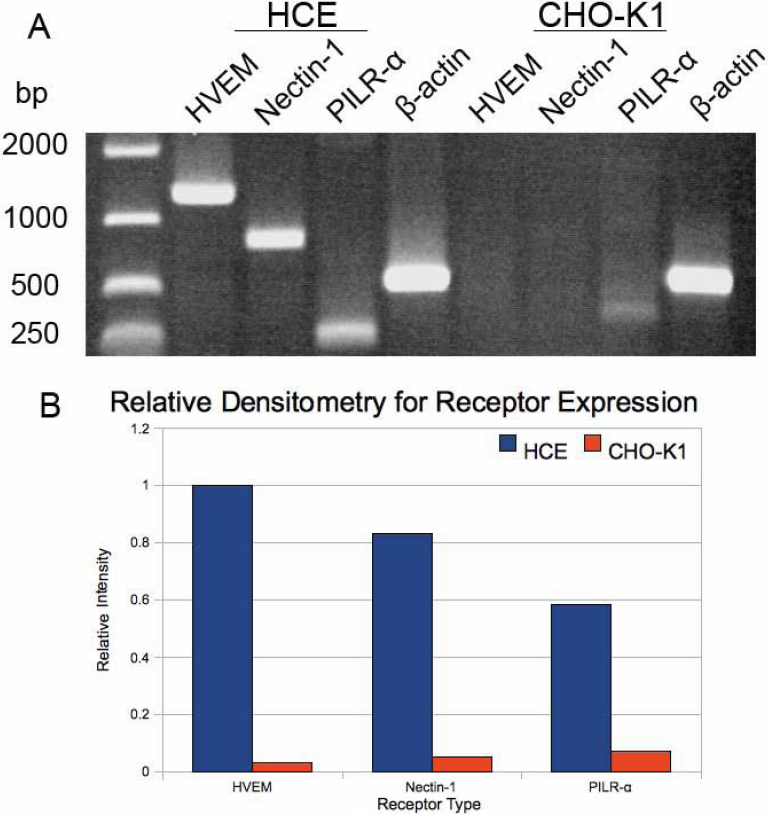
Quantitative RT–PCR analysis for entry receptor expression. Total RNA was isolated from HCE and CHO-K1 cells and converted to cDNA and analyzed by PCR for the receptors as indicated. A house keeping gene, β-actin (*ACTB*), was used as a control (**A**). Densitometry analysis was performed using the NIH ImageJ software (Version 1.43) and data for relative intensity was plotted as a histogram (**B**).

**Figure 5:**
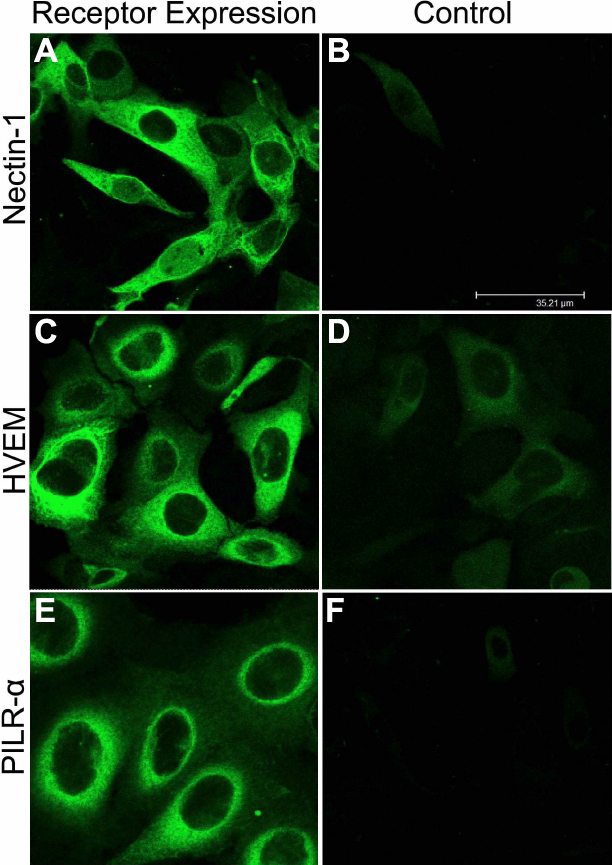
Immunofluorescence imaging of receptors on HCE cell membrane. Images shown were taken using the FITC filter of confocal microscope (Leica SP20). Cells were blocked for 90 min, washed, and then either mock treated with buffer alone (**B**, **D**, **F**) or treated with primary antibodies for Nectin-1 (**A**), HVEM (**C**), and PILR-alpha (**E**). Images were taken after the incubation of HCE cells with FITC-conjugated secondary antibodies. Staining of cells with green demonstrate receptor expression.

**Figure 6:**
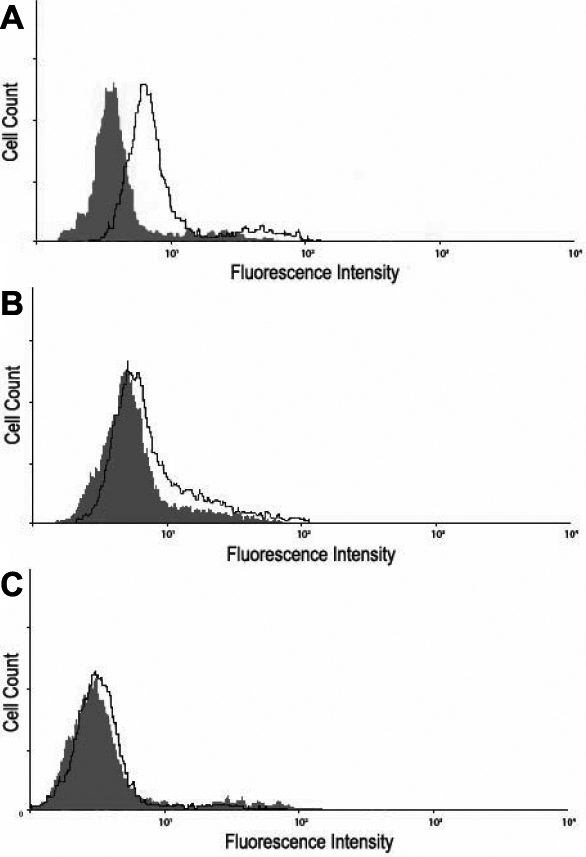
Flow cytometry analysis of cell-surface receptor expression. Expression was detected by Fluorescence-activated cell sorter (FACS) analysis. Cells were treated with primary antibodies to Nectin-1 (**A**), HVEM (**B**), or PILR-alpha (**C**). HCE cells stained only with FITC-conjugated secondary antibody were used as background controls and are shown as the dark gray in the figure.

To explore the molecular basis for entry, we decided to examine entry receptor expression by quantitative RT–PCR. It was evident that genes for HVEM, Nectin-1 and PILRalpha were clearly expressed by HCE cells (Figure 4A). Densitometry confirmed gene expression of all three receptors as well (Figure 4B). The control, CHO-K1 cells, showed possible expression of PILRalpha but not HVEM or nectin-1. As expected, both cell-types expressed β-actin. The results from CHO-K1 cells are consistent with previous findings [21]. Next, we decided to verify the gene expression results by protein expression analysis using immunofluorescence and flow-cytometry. As shown in Figure 5A,C,E, respectively, HCE cells express nectin-1, HVEM, and PILR-α along the cell membrane. Cells treated with only FITC-conjugated secondary antibody as negative controls (Figure 5B,D,F, respectively) did not show fluorescence and likewise, isotype control experiments also did not result in any signals (data not shown). The expression of all three receptors was also confirmed by flow cytometry and is demonstrated in Figure 6. While the intensity of positive cells in the x-axis is somewhat low, these results were reproducible and confirm our findings from immunofluorescence (Figure 5). The expression of nectin-1 was most abundant, followed by HVEM and PILR-α.

### Antibody blocking and down-regulation of receptors

**Figure 7:**
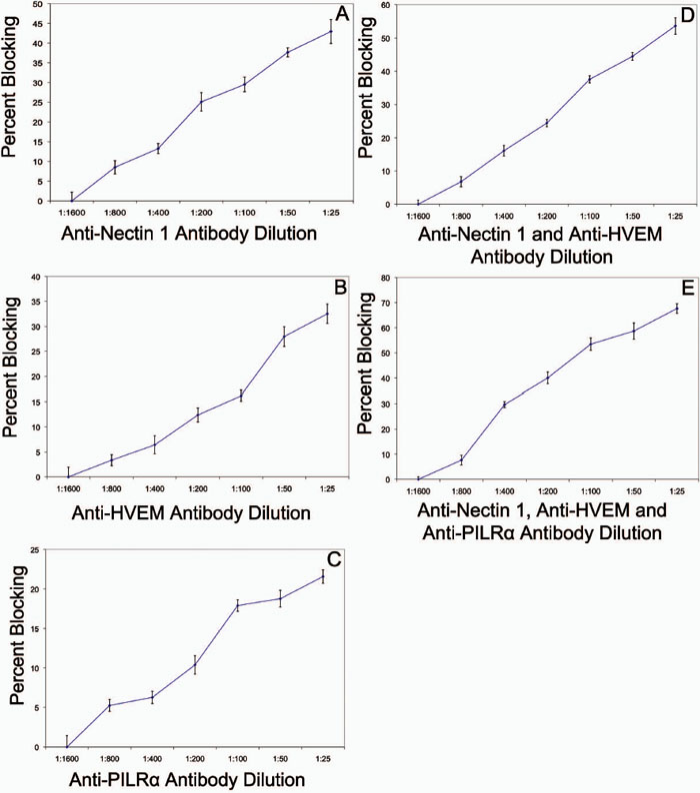
Antibodies to the major receptors block HSV-1 entry. Monolayers of cells plated in 96-well culture dishes were incubated with serial dilutions of primary antibodies to Nectin-1, HVEM, and PILR-α for 2 h. Cells were then exposed to HSV-1(KOS)gL86 virus and viral entry was measured 6 h post-infection using a spectrophotometer at an OD of 410 nm. The ratio of entry between cells treated and those untreated are reported as percent blocking. Combinations of antibodies that block these receptors were also studied. Data shown are the means of triplicate determinations (±SD). Anti-nectin-1 (**A**), anti-HVEM (**B**), anti-PILR-α (**C**), a combination of anti-nectin-1 and anti-HVEM antibodies (**D**) and a combination of all three antibodies (**E**).

Reduction of viral entry with blocking by receptor-specific antibodies would indicate the role of those receptors in pathogenesis. The results shown in Figure 7 indicate that there is remarkably diminished entry when any of the receptors (nectin-1, HVEM, and PILR-α) are blocked. Data was recorded as percent blocking, meaning the percentage that entry was reduced compared to controls. Maximum percent blocking of approximately 45%, 30%, and 20% were seen when the cells were treated with anti-nectin-1 (Figure 7A), anti-HVEM (Figure 7B), and anti-PILR-α (Figure 7C) antibodies, respectively. Further reduction was seen when multiple receptors were blocked – there was approximately 50% blocking when the anti-nectin-1 and anti-HVEM antibodies were used in coordination (Figure 7D) and nearly 70% blocking when all three antibodies were used (Figure 7E). Absolute numbers for blocking are shown in Table 1.

**Table 1:** Absolute numbers for antibody blocking assay.

**Dilution factor**	**Untreated**	**Anti-nectin Ab**	**Anti-HVEM Ab**	**Anti-PILRα Ab**	**Anti-nectin and Anti-HVEM**	**Anti-nectin/ Anti-HVEM/ Anti-PILRα Ab**
1:1600	0.79±0.01	0.79±0.02	0.75±0.01	0.78±0.01	0.77±0.01	0.77±0.01
1:800	0.79±0.03	0.72±0.01	0.72±0.01	0.74±0.01	0.72±0.01	0.71±0.01
1:400	0.77±0.02	0.68±0.01	0.7±0.01	0.74±0.01	0.65±0.01	0.54±0.01
1:200	0.78±0.01	0.59±0.02	0.66±0.01	0.7±0.01	0.58±0.01	0.46±0.02
1:100	0.77±0.03	0.55±0.01	0.63±0.01	0.64±0.01	0.48±0.01	0.36±0.02
1:50	0.78±0.02	0.49±0.01	0.54±0.01	0.64±0.01	0.43±0.01	0.32±0.02
1:25	0.78±0.02	0.45±0.02	0.51±0.01	0.62±0.01	0.36±0.02	0.25±0.01

### Nectin-1 expression may reduce re-infection

**Figure 8:**
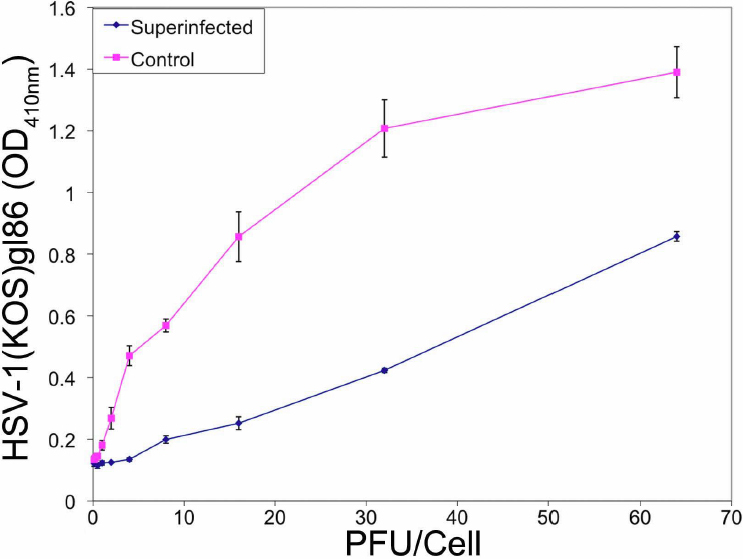
Superinfected cells demonstrate less viral entry. Monolayers of cells plated in 96-well culture dishes were treated with a primary viral dose of HSV-1(KOS) at an MOI of 0.01. After 48 h of incubation at 37 °C, cell were treated with serial dilution of a secondary viral dose of HSV-1(KOS)gL86. Viral entry was measured using a spectrophotometer at an OD of 410 nm. Cells solely infected with only one dose of HSV-1(KOS)gL86 were used as control.

**Figure 9:**
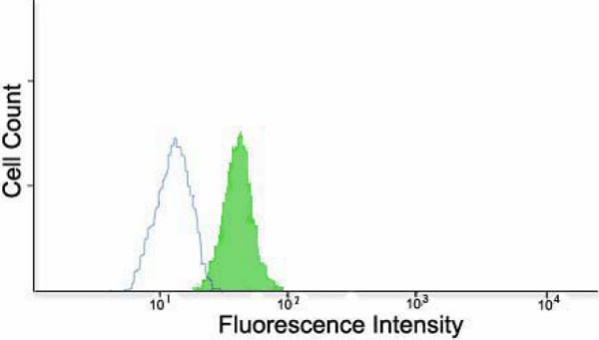
Cells demonstrate a decreased level of Nectin-1 receptor expression after primary infection. Monolayers of HCE cells plated on a 6-well tissue culture dish were treated with a viral dose at an MOI of 0.01 and incubated for 48 h at 37 °C. Cells were then treated with MTT to distinguish live from dead cells. FACS was then performed to test levels of Nectin-1 on living infected cells tagged with red fluorescent protein. HCE cells untreated with a viral dose were used as a control and are shown as the green shade.

The re-infection experiment was performed to indicate whether HSV-1 entry was affected by a previous low-level infection. The results shown in Figure 8 demonstrate a decrease in HSV-1 (gL-86) entry into cells that have already undergone a primary infection with wild-type HSV-1 (KOS) (superinfected) compared to control, which were not exposed to HSV-1(KOS) before HSV-1(gL86) expression. To analyze the percentage of cells that were infected at this time point (48 h after primary infection), cells were stained for β-galactosidase expression from the secondary viral genome [27]. The number of cells infected in by secondary reporter virus averaged at 27±4% (data not shown) compared to about 100% infected control cells. To gain insight into a possible mechanism for the decrease in infection, flow cytometry was performed post-primary infection to determine nectin-1 expression compared to control. The results in Figure 9 show a decrease in the level of nectin-1 expression on living cells 48 h post-primary infection compared to living control cells that underwent no primary infection and instead were simply incubated for 48 h. This result, however, has to be seen in the light of the observation that overall expression of nectin-1 goes up significantly from about 24 h of incubation (Figure 6) to after 48 h of incubation. Thus, a prior infection by a HSV-1 strain can reduce the nectin-1 expression relative to uninfected cells.

## Discussion

HSV-1 can infect the cornea and cause keratitis – an often treatable but potentially visually debilitating disease that can progress to dendritic ulceration as well as corneal neovascularization and scarring through repeated episodes [1]. HSV-1 epithelial keratitis predisposes to infection of the deeper layers of the cornea [1,2,8]. The ability of HSV-1 to develop latent infection in trigeminal ganglia creates the threat of ocular disease to affected patients throughout their lives [8,25-27].

While the cornea represents the gateway to infection in other parts of the eye, little is known of the cellular and molecular mediators of viral entry into corneal epithelial cells. As a preliminary step toward understanding this process, our study provides knowledge on specific features of HSV-1 entry. We first demonstrated that HSV-1 can successfully enter and infect the HCE cell line, and that the infection process relies on the receptors nectin-1, HVEM, and PILR-α, located on the cell membrane. It was noted that while HCE cells express all three receptors, cell types show different expression patterns [28,29].

Importantly, corneal fibroblasts do not express nectin-1 [30]. This may provide some insight into the pathogenesis of herpetic keratitis; in addition to known features of ocular herpes pathogenesis, unique virus-cell interactions in the entry process may occur. HSV-1 entry into trabecular meshwork cells is primarily facilitated by HVEM, whereas in conjunctival epithelial cells it is aided by nectin-1 and HVEM [25,28,31].

The importance of all three receptors (nectin-1, HVEM, and PILR-α) for HSV-1 entry was confirmed via antibody-blocking, which demonstrated decreased viral entry when one or more receptor was blocked. Maximum reduction of viral entry was seen when multiple receptors were blocked. The entry graphs did not plateau and some entry was still observed when all three antibodies were used with maximal blocking of 70%. This suggests one of the following: antibody blocking was incomplete which would otherwise have prevented all viral entry; 3-O sulfated heparan sulfate plays a role in entry into HCE cells; there is/are unknown mechanism(s) involved.

Our entry results demonstrated that nectin-1 was the most significant receptor for entry, followed by HVEM and then PILR-α. Given current knowledge of the role of PILR-α in HSV infection, it may more accurately be described as a co-receptor that aids other primary receptors in entry [21]. Entry was also shown to be pH dependent and thus may utilize a vesicular form of entry that relies on acidification [18].

The pathogenesis of corneal epithelial infection by HSV is not well understood, which includes periods of active virus during which cellular entry and spread occurs. With episodes lasting several days, we were interested in re-infection in the HCE cell line. Our study demonstrated a lower efficiency of secondary viral infection at 24 to 48 h. Therefore, this is further evidence that virus-cell interactions may be important in the infection process. Due to limitations of our in vitro system we were unable to investigate substantially longer time points.

To determine whether re-infection correlated with a change in receptor expression, flow cytometry was performed to test for levels of nectin-1, which was the primary receptor in viral entry. The result that average nectin-1 expression was reduced after the initial infection is an observation that requires further investigation. The implications of this decrease including whether it plays a role in the trends of re-infection are future areas of interest.

Studies are also required to further investigate the molecular mechanisms of viral entry in the corneal epithelium. As mentioned, our result that nectin-1 is the primary receptor for entry is in contrast to what is known for corneal fibroblasts [30]. This is an important consideration for future therapeutic modalities targeting entry mechanisms. For instance, one may postulate that blocking nectin-1 may attenuate corneal epithelial infection, whereas for stromal infection this would be less important. The presence of nectin-1 in murine corneal epithelium and other ocular tissues has been described previously [32]. With this in mind, the roles of nectin-1, HVEM, and PILR-α in corneal epithelial infection also need to be assessed using in vivo models. The finding of pH dependency of HSV-1 entry into HCE cells must also be further probed as this suggests a possible endocytic mode of entry. The possibility of a pH-altering treatment could also be studied in vivo, although ocular toxicity would be an important limiting factor. Finally, reduced secondary viral entry and changes in expression of entry receptors after exposure to virus may influence pathogenesis. As HSV-1 infection of the cornea remains a significant cause of morbidity, this study provides important insights to guide future work.

## References

[1] Toma HS Murina AT Areaux RG Jr Neumann DM Bhattacharjee PS Foster TP Kaufman HE Hill J Ocular HSV-1 latency, reactivation and recurrent disease. Semin Ophthalmol 2008 23 249 73 18584563 10.1080/08820530802111085

[2] Liesegang TJ Herpes simplex virus epidemiology and ocular importance. Cornea 2001 20 1 13 11188989 10.1097/00003226-200101000-00001

[3] Knickelbein JE Hendricks RL Charukamnoetkanok P Management of herpes simplex virus stromal keratitis: an evidence-based review. Surv Ophthalmol 2009 54 226 34 19298901 10.1016/j.survophthal.2008.12.004

[4] Kaye S Choudhary A Herpes simplex keratitis. Prog Retin Eye Res 2006 25 355 80 16807055 10.1016/j.preteyeres.2006.05.001

[5] Cook SD Brown SM Herpes simplex virus type 1 persistence and latency in cultured rabbit corneal epithelial cells, keratocytes, and endothelial cells. Br J Ophthalmol 1986 70 642 50 3019382 10.1136/bjo.70.9.642 PMC1040792

[6] Morris DJ Cleator GM Klapper PE Cooper RJ Biney EO Dennett C Marcyniuk B Detection of herpes simplex virus DNA in donor cornea culture medium by polymerase chain reaction. Br J Ophthalmol 1996 80 654 7Tullo AB 8795381 10.1136/bjo.80.7.654 PMC505563

[7] Wilhelmus KR Coster DJ Donovan HC Falcon MG Jones BR Prognostic Indicators of Herpetic Keratitis: Analysis of a Five-Year Observation Period after Corneal Ulceration. Arch Ophthalmol 1981 99 1578 82 6793030 10.1001/archopht.1981.03930020452009

[8] Herpetic Eye Disease Study Group Acyclovir for the prevention of recurrent herpes simplex virus eye disease. N Engl J Med 1998 339 300 6 9696640 10.1056/NEJM199807303390503

[9] Panda A Vanathi M Kumar A Dash Y Priya S Corneal graft rejection. Surv Ophthalmol 2007 52 375 96 17574064 10.1016/j.survophthal.2007.04.008

[10] Garcia DD Shtein RM Musch DC Elner VM Herpes simplex virus keratitis: histopathologic neovascularization and corneal allograft failure. Cornea 2009 28 963 5 19724220 10.1097/ICO.0b013e31819c4e55 PMC2936268

[11] Zheng X Reactivation and donor-host transmission of herpes simplex virus after corneal transplantation. Cornea 2002 21 S90 3 12484706 10.1097/01.ico.0000263126.76392.cf

[12] Nicholls SM Shimeld C Easty DL Hill TJ Recurrent herpes simplex after corneal transplantation in rats. Invest Ophthalmol Vis Sci 1996 37 425 35 8603848

[13] Remeijer L Duan R Dun J Bettink M Prevalence and clinical consequences of herpes simplex type 1 DNA in human cornea tissue. J Infect Dis 2009 200 11 9 19476433 10.1086/599329

[14] Hill JM Clement C Herpes simplex virus type 1 DNA in human corneas: what are the virological and clinical implications? J Infect Dis 2009 200 1 4 19476431 10.1086/599330 PMC2874965

[15] Spear PG Herpes simplex virus: receptors and ligands for cell entry. Cell Microbiol 2004 6 401 10 15056211 10.1111/j.1462-5822.2004.00389.x

[16] Shukla D Spear PG Herpesviruses and heparan sulfate: an intimate relationship in aid of viral entry. J Clin Invest 2001 108 503 10 11518721 10.1172/JCI13799 PMC209412

[17] Scanlan PM Tiwari V Bommireddy S Shukla D Cellular expression of gH confers resistance to herpes simplex virus type-1 entry. Virology 2003 312 14 24 12890617 10.1016/s0042-6822(03)00176-4

[18] Nicola AV McEvoy AM Straus SE Roles for endocytosis and low pH in herpes simplex virus entry into HeLa and Chinese hamster ovary cells. J Virol 2003 77 5324 32 12692234 10.1128/JVI.77.9.5324-5332.2003 PMC153978

[19] Montgomery RI Warner MS Lum BJ Spear PG Herpes simplex virus-1 entry into cells mediated by a novel member of the TNF/NGF receptor family. Cell 1996 87 427 36 8898196 10.1016/s0092-8674(00)81363-x

[20] Shukla D Liu J Blaiklock P Shworak NW Bai X Esko JD Cohen GH Eisenberg RJ Rosenberg RD Spear PG A novel role for 3-O-sulfated heparan sulfate in herpes simplex virus 1 entry. Cell 1999 99 13 22 10520990 10.1016/s0092-8674(00)80058-6

[21] Satoh T Arii J Suenaga T Wang J Kogure A Uehori J Arase N Shiratori I Tanaka S Kawaguchi Y Spear PG Lanier LL Arase H PILRalpha is a herpes simplex virus-1 entry coreceptor that associates with glycoprotein B. Cell 2008 132 935 44 18358807 10.1016/j.cell.2008.01.043 PMC2394663

[22] Mott KR Bresee CJ Allen SJ BenMohamed L Wechsler SL Ghiasi H Level of herpes simplex virus type 1 latency correlates with severity of corneal scarring and exhaustion of CD8+ T cells in trigeminal ganglia of latently infected mice. J Virol 2009 83 2246 54 19091870 10.1128/JVI.02234-08 PMC2643740

[23] Araki-Sasaki K Ohashi Y Sasabe T Hayashi K Watanabe H Tano Y Handa H An SV40-immortalized human corneal epithelial cell and its characterization. Invest Ophthalmol Vis Sci 1995 36 614 21 7534282

[24] Yoshimori T Yamamoto A Moriyama Y Futai M Tashiro Y Bafilomycin A1, a specific inhibitor of vacuolar-type H(+)-ATPase, inhibits acidification and protein degradation in lysosomes of cultured cells. J Biol Chem 1991 266 17707 12 1832676

[25] Akhtar J Kovacs M Oh M Tiwari V Jani A HVEM and nectin-1 determine herpes simplex virus 1 (HSV-1) entry into human conjunctival epithelium. Invest Ophthalmol Vis Sci 2008 49 4026 35 18502984 10.1167/iovs.08-1807 PMC2569872

[26] Pepose JS Keadle TL Morrison LA Ocular herpes simplex: changing epidemiology, emerging disease patterns, and the potential of vaccine prevention and therapy. Am J Ophthalmol 2006 141 547 57 16490506 10.1016/j.ajo.2005.10.008

[27] Shukla SY Singh Y Shukla D Role of nectin-1, HVEM, and PILR-alpha in HSV-2 entry into human retinal pigment epithelial cells. Invest Ophthalmol Vis Sci 2009 50 2878 87 19234349 10.1167/iovs.08-2981 PMC3686556

[28] Tiwari V Clement C Scanlan PM Yue BY Shukla D A role for HVEM as the receptor for herpes simplex virus-1 entry into primary human trabecular meshwork cells. J Virol 2005 79 13173 9 16189018 10.1128/JVI.79.20.13173-13179.2005 PMC1235852

[29] Taylor JM Lin E Susmarski N Yoon M Zago A Ware CF Pfeffer K Miyoshi J Takai Y Spear PG Alternative entry receptors for herpes simplex virus and their roles in disease. Cell Host Microbe 2007 2 19 28 18005714 10.1016/j.chom.2007.06.005 PMC2083283

[30] Tiwari V Clement C Xu D Valyi-Nagy T Yue B Liu J Shukla D Role of 3-O-sulfated heparan sulfate as the receptor for herpes simplex virus type 1 entry into primary human corneal fibroblasts. J Virol 2006 80 8970 80 16940509 10.1128/JVI.00296-06 PMC1563926

[31] Farooq AV Valyi-Nagy T Shukla D Mediators and mechanisms of herpes simplex virus entry into ocular cells. Curr Eye Res 2010 35 445 50 20465436 10.3109/02713681003734841 PMC2902162

[32] Valyi-Nagy T Sheth V Clement C Tiwari V Scanlan P Kavouras JH Leach L Guzman-Hartman G Dermody TS Shukla D Herpes simplex virus entry receptor nectin-1 is widely expressed in the murine eye. Curr Eye Res 2004 29 303 9 15590476 10.1080/02713680490516756

